# Near-infrared luminescent metallacrowns for combined *in vitro* cell fixation and counter staining†
†Electronic supplementary information (ESI) available: Brightfield and fluorescence cell imaging, excitation and emission spectra. See DOI: 10.1039/c7sc01872j



**DOI:** 10.1039/c7sc01872j

**Published:** 2017-08-08

**Authors:** Ivana Martinić, Svetlana V. Eliseeva, Tu N. Nguyen, Frédéric Foucher, David Gosset, Frances Westall, Vincent L. Pecoraro, Stéphane Petoud

**Affiliations:** a Centre de Biophysique Moléculaire , CNRS , UPR 4301 , 45071 Orléans Cedex 2 , France . Email: svetlana.eliseeva@cnrs.fr ; Email: stephane.petoud@inserm.fr; b Department of Chemistry , Willard H. Dow Laboratories , University of Michigan , 930 N. University Ave , Michigan 48109 , USA . Email: vlpec@umich.edu

## Abstract

Cell fixation is an essential approach for preserving cell morphology, allowing the targeting and labelling of biomolecules with fluorescent probes. One of the key requirements for more efficient fluorescent labelling is the preservation of cell morphology, which usually requires a combination of several fixation techniques. In addition, the use of a counter stain is often essential to improve the contrast of the fluorescent probes. Current agents possess significant limitations, such as low resistance toward photobleaching and sensitivity to changes in the microenvironment. Luminescent Ln^3+^ ‘encapsulated sandwich’ metallacrowns (MCs) overcome these drawbacks and offer complementary advantages. In particular, they emit sharp emission bands, possess a large difference between excitation and emission wavelengths and do not photobleach. Herein, MCs formed with pyrazinehydroxamic acid (Ln^3+^[Zn(ii)MC_pyzHA_], Ln^3+^ = Yb, Nd) were used, combined with near-infrared (NIR) counter staining and fixation agents for HeLa cells upon an initial five minute exposure to UV-A light. The validity and quality of the cell fixation were assessed with Raman spectroscopy. Analysis of the NIR luminescence properties of these MCs was performed under different experimental conditions, including in a suspension of stained cells. Moreover, the high emission intensity of Ln^3+^[Zn(ii)MC_pyzHA_] in the NIR region allows these MCs to be used for imaging with standard CCD cameras installed on routine fluorescence microscopes. Finally, the NIR-emitting Ln^3+^[Zn(ii)MC_pyzHA_] compounds combine, within a single molecule, features such as cell fixation and staining abilities, good photostability and minimal sensitivity of the emission bands to the local microenvironment, and they are highly promising for establishing the next generation of imaging agents with a single biodistribution.

## Abstract 2

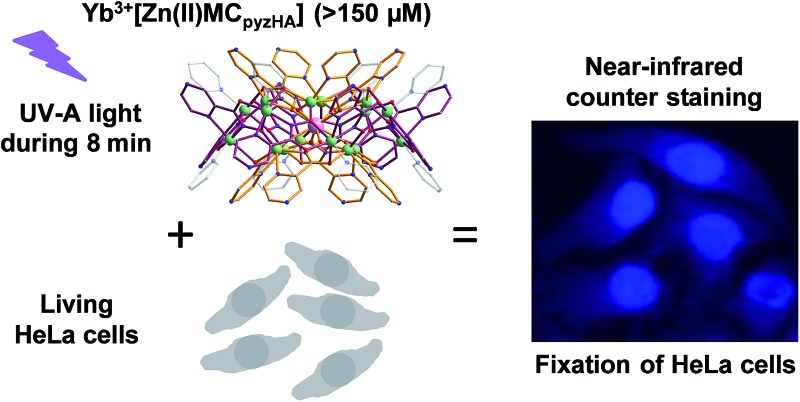
Combined cell fixation and near-infrared counter staining was achieved using Ln^III^/Zn^II^ MCs with pyrazinehydroxamic acid upon illumination with UV-A light.

## Introduction

The field of optical biological imaging has grown explosively in recent years due to blooming technological advances related to detection techniques and image treatment. Fluorescent probes allow the visualization and/or quantification of biological objects or events with high detection sensitivity and resolution at the cellular level.^1^ Due to the limited number of fluorescent probes with high targeting efficiencies for specific antigens in living cells, cell fixation is the best approach in many research cases.^2^ Cell fixation preserves the cellular and tissue morphology in a ‘life-like state’. This process is a critical first step prior to cell staining, which is usually performed with selective fluorescent probes conjugated with targeting moieties, *e.g.* peptides, carbohydrates or antibodies.

Several fixation techniques have been reported and applied.^3^ The most common examples involve crosslinking with the fixative paraformaldehyde, preferred for protein targeting, while alcohol-based precipitating fixatives are best suited for the study of RNA-containing molecules.^2^ Nevertheless, none of these techniques/fixation agents are ideal and their combination is often required to obtain satisfactory results, thus complicating the experiments and inducing artifacts in the cellular sample to be analysed. Moreover, for each fluorescent probe, it is necessary to individually optimize the experimental conditions in order to allow unhindered targeting access to the specific desired cell compartments. Finally, in order to increase the contrast obtained with specific fluorescent probes after the primary staining step, a counter stain often needs to be applied. As a drawback, this operation may result in an undesirable overlap between the excitation and emission bands of the counter stain and the specific targeting probe, creating a reabsorption effect, which is detrimental for quantification.

Due to the strong absorption of UV and visible light by tissue components,^4,5^ the shifting of the excitation and emission wavelengths toward lower energy is in strong demand.^6–10^ In addition, interest in the development of fluorescent probes emitting in the near-infrared (NIR) region is rapidly increasing due to the improvement of the signal-to-noise ratio and the detection sensitivity through the minimization of autofluorescence signals.

The design of novel agents that combine the fluorescent labelling of cells with fixation is highly desirable to avoid the perturbation of biological systems caused by using multiple reagents. A combined fixation/staining agent obtained with a visible-emitting nuclear stain (Hoechst 33342) has been reported previously by Davis and Bardeen.^11^ In these experiments, the crosslinking of histone proteins with DNA has been observed for chromatin stained with the fluorescent agent upon excitation with UV-A light.^11^ Nevertheless, to date, there is no report for the combined fixation and fluorescent labelling of a whole cell with a single agent.

The majority of commercial fluorescent probes rely on organic dyes,^6^ such as derivatives of fluoresceins/rhodamines, bodipys, cyanines, porphyrins or phthalocyanines. These organic stains possess drawbacks such as limited chemical stabilities, broad emission bands, small Stokes shifts and fast photobleaching, which result in difficulties in the quantification of the emission intensities. The latter can be reduced in some cases by using anti-fade reagents,^12^ which allow the preservation of the fluorescence signal intensity over a longer period of time, and/or by use of specialized software for image treatment. To avoid this extra step and remove the risk of false negatives, inorganic optical probes, such as quantum dots (QDs) or lanthanide complexes, can be used. QDs exhibit superior photophysical properties over organic fluorophores, such as higher quantum yield values, improved photostability and narrower emission bands.^13^ However, they are still affected by some drawbacks, such as the blinking effect and the toxicity associated with their typically heavy atom components.^14^

Lanthanide(iii)-based complexes are a class of luminescent compound that possess fascinating and unique optical properties due to the electronic structure of the lanthanide(iii) cations (Ln^3+^) they incorporate.^15,16^ More specifically, Ln^3+^ cations exhibit f–f emission bands from the visible to the NIR range and have a number of complementary properties with respect to the fluorescent probes: sharp emission bands that are highly insensitive to the microenvironment, large energy differences between the absorption and emission bands and high resistance toward photobleaching (allowing long term or repetitive quantitative experiments).^15,17^ Most of the free Ln^3+^ cations exhibit very low molar absorption coefficients due to the parity-forbidden selection rules, which result in weak emission intensities of the f–f transitions upon direct excitation.^18,19^ This limitation has been overcome by taking advantage of the ‘antenna effect’, *i.e.* locating an appropriate chromophoric group, an ‘antenna’, in sufficiently close proximity to the Ln^3+^ ion.^20^ Several Ln^3+^-based complexes and nanomaterials have been used for cell imaging to date, under either one photon^21,22^ or two photon excitation.^23,24^

**Fig. 1:**
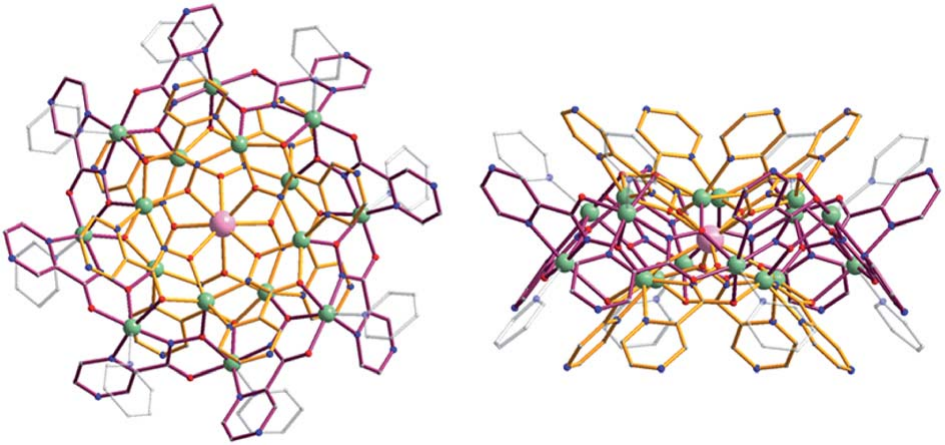
Representation of the crystal structure of Yb^3+^[Zn(ii)MC_pyzHA_] obtained by X-ray diffractometry on a single crystal: (left) top-down view and (right) side-view. Color scheme: light green, Zn^2+^; light purple, Yb^3+^; red, O; blue, N; gray, C; bronze, [12-MC_Zn(II),pyzHA_-4]; dark purple, [24-MC_Zn(II),pyzHA_-8]. Hydrogen atoms have been omitted for clarity.^27^

We have reported previously the design, synthesis, characterization and luminescence properties of Ln^3+^ ‘encapsulated sandwich’ metallacrown (MC) complexes based on Zn^2+^ ions and bivalent aromatic hydroximate ligands (L^2–^), derivatives of picoline- or quinolinehydroxamic acids.^25,26^ These MCs, with the general composition Ln^3+^[12-MC_Zn(II),L_-4]_2_[24-MC_Zn(II),L_-8] (Ln^3+^ = Nd, Er, Yb), exhibit outstanding photophysical properties in the NIR region with high quantum yield values (in comparison to NIR emitting Ln^3+^-based complexes containing C–H bonds) and long luminescence lifetimes in the solid state and in methanol solutions. This was possible to achieve due to the efficient Ln^3+^ sensitization and strong protection against non-radiative deactivation pathways (resulting from the overtones of high energy C–H, N–H and O–H vibrations located in solvent molecules and ligands close to Ln^3+^) in Ln^3+^/Zn^2+^ MCs. However, since the water solubility of these complexes is limited, they are not well-suited for direct applications in biological media. In order to increase the water solubility of these complexes while retaining their desired photophysical properties, we have developed MCs possessing a similar structure type using pyrazinehydroxamic acid (H_2_pyzHA) to prepare Ln^3+^[12-MC_Zn(II),pyzHA_-4]_2_[24-MC_Zn(II),pyzHA_-8] (Ln^3+^[Zn(ii)MC_pyzHA_], Ln^3+^ = Yb, Nd) (Fig. 1). These water soluble MCs exhibit intense NIR emission and high photostability, and are able to preferentially label necrotic cells.^27^

Herein, we explore a novel biological application of Ln^3+^[Zn(ii)MC_pyzHA_] (Ln^3+^ = Yb, Nd) MCs as combined agents to induce cell fixation upon exposure to UV-A light and, simultaneously, as photostable stains for whole-cell visualization by NIR optical microscopy. We have performed brightfield and Raman microscopy and Raman spectroscopy to demonstrate that the morphology of cells is not affected by the use of these staining agents. In addition, a study into the variation of the photophysical properties (emission and excitation spectra, luminescence quantum yields and lifetimes) of these MCs, under different experimental conditions including when located inside HeLa cells, has been performed and is described herein.

## Experimental section

### Synthesis of Ln^3+^[Zn(ii)MC_pyzHA_] (Ln^3+^ = Yb, Nd)

The Ln^3+^[Zn(ii)MC_pyzHA_] complexes (Ln^3+^ = Yb, Nd) were synthesized according to the previously reported procedure.^27^ The MCs in crystalline form were isolated as triflate salts, with the general composition [Zn_16_Ln(pyzHA)_16_(py)_8_](OTf)_3_(H_2_O)_12_, by filtration and dried in air. For further experiments, 5 mM stock solution of Ln^3+^[Zn(ii)MC_pyzHA_] in water was prepared and further diluted in cell culture media to the desired concentration.

### Cell fixation with Ln^3+^[Zn(ii)MC_pyzHA_] and NIR epifluorescence microscopy

The HeLa (human cervical carcinoma cells) cell line obtained from ATCC (Molsheim, France) was cultured in Dulbecco’s Modified Eagle’s Medium (DMEM), supplemented with 10% heat-inactivated fetal bovine serum (FBS, purchased from Sigma, F7524), 1% of 100× non-essential amino acid solution (Sigma, M7145), 1% of l-glutamine (GlutaMAX) and 1% of streptomycin/penicillin antibiotics (Sigma, P4333). Cells were seeded in an 8-well Lab Tek Chamber coverglass (Nunc, Dutsher S.A., Brumath, France) at a density of 6 × 10^4^ cells per well and cultured at 37 °C in a 5% humidified CO_2_ atmosphere. After 24 h, the cell culture medium was removed, the cells were washed twice with Opti-MEM reduced serum medium (at room temperature), pre-incubated with solutions of 15 μM, 30 μM, 60 μM and 150 μM Ln^3+^[Zn(ii)MC_pyzHA_] in Opti-MEM medium (supplemented with 2% of FBS at 37 °C in a 5% CO_2_ atmosphere) for 15 min, illuminated with UV-A light (377 nm bandpass 50 nm filter, 8.6 mW cm^–2^ power density) for 5, 8 or 10 min, followed by incubation for 1 h (in order to allow the internalization of the complex), then washed with Opti-MEM medium and incubated with 3 μM propidium iodide (PI) for 5 min. Prior to epifluorescence imaging, the cells were washed twice with Opti-MEM (at room temperature) in order to remove any non-specifically bound Ln^3+^[Zn(ii)MC_pyzHA_]. The obtained cells were observed with a Zeiss Axio Observer Z1 fluorescence inverted microscope (Zeiss, Le Pecq, France) equipped with an EMCCD Photometrics Evolve 512 (Roper Scientific) camera or a Hamamatsu ORCA-R2 high-resolution CCD camera. The Zeiss HXP 120 white light source was used in combination with the following filter cubes: (i) a 447 nm bandpass 60 nm filter for the excitation, and a longpass 805 nm or 996 nm bandpass 70 nm filter to monitor the Yb^3+^ emission; (ii) a 377 nm bandpass 50 nm filter for the excitation, and a longpass 785 nm filter to discriminate the Nd^3+^ emission; (iii) a 550 nm bandpass 25 nm filter for the excitation, and a 605 nm bandpass 70 nm filter for the emission of PI in the visible range.

### Cell fixation using standard techniques

Paraformaldehyde (PFA) diluted in a phosphate buffered saline (PBS) solution was used for the fixation of cells. HeLa cells were incubated with a 4% PFA solution for 30 min at room temperature, followed by a rinsing step with PBS (3×), and were stored at +4 °C until required. Cell fixation with methanol was obtained upon incubation of HeLa cells with ice cold methanol (100%) at –20 °C for 6 min. Such fixation was followed by PBS rinsing for 5 min for each of the washing steps (3×).

### Confocal microscopy

For confocal microscopy experiments, cells were prepared in a similar way as those for epifluorescence microscopy. The cells were observed with confocal laser scanning microscopy (CLSM) on a Zeiss Axiovert 200M microscope equipped with a LSM 510 Meta scanning device (Zeiss, France). Yb^3+^[Zn(ii)MC_pyzHA_] was excited with an argon laser at 458 nm and the emission signal was collected from 499 nm to 799 nm. A 63× Plan-Apochromat objective was used for these experiments.

### Photophysical properties

Photophysical data were collected on samples (solutions or suspensions of cells) placed into 2.4 mm i.d. quartz capillaries. For the preparation of the suspension of HeLa cells, the HeLa cells were fixed with Yb^3+^[Zn(ii)MC_pyzHA_] or Nd^3+^[Zn(ii)MC_pyzHA_] complexes under optimized conditions (150 μM concentration and 8 min of illumination using a Zeiss HXP 120 white light source selected with a 377 nm bandpass 50 nm filter). The fixed cells were detached by incubation with a 0.25% trypsine solution (diluted in PBS) at 37 °C for 30 min, washed, suspended in Opti-MEM cell culture medium supplemented with 2% FBS and placed in quartz capillaries. Emission and excitation spectra were measured on a custom-designed Horiba Scientific Fluorolog 3 spectrofluorimeter equipped with either a visible photomultiplier tube (PMT) (220–850 nm, R928P, Hamamatsu) or a NIR PMT (940–1650 nm, H10330-75, Hamamatsu). The excitation and emission spectra were corrected for the instrumental functions. Luminescence lifetimes were determined under excitation at 355 nm provided by a Nd:YAG laser (YG 980, Quantel). Signals were detected in the NIR region with the help of a Hamamatsu H10330-75 PMT. Output signals from the detector were fed into a 500 MHz bandpass digital oscilloscope (TDS 754C, Tektronix) and transferred to a PC for data processing with the program Origin 8®. The luminescence lifetimes are averages of at least three independent measurements. Quantum yields were determined with the Fluorolog 3 spectrofluorimeter based on an absolute method using an integration sphere (Model G8, GMP SA, Renens, Switzerland). Each sample was measured several times under comparable experimental conditions, varying the position of the samples. The estimated experimental error for the quantum yield determination is ∼10%.

### Confocal Raman spectroscopy

Raman spectroscopy mapping was performed with a WITec Alpha 500RA system using a green Nd:YAG frequency-doubled laser (532 nm wavelength). The laser power was set to below 20 mW to prevent the heating of the cells. The experiments were performed in Opti-MEM using a 60× water immersion objective (LUMPLFLN 60XW, Olympus). The averaged spectra of different cells/compartments were extracted from the maps.

## Results and discussion

**Fig. 2:**
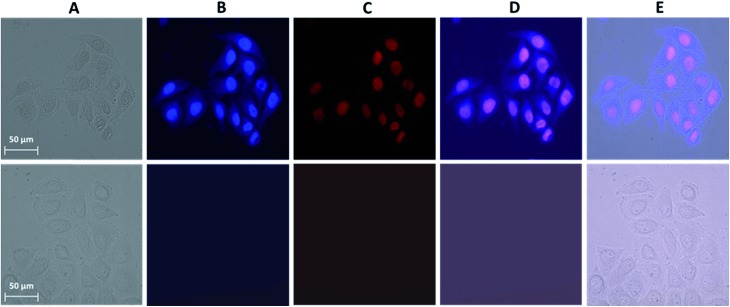
Images obtained from epifluorescence microscopy experiments performed on HeLa cells. (Top) Incubated with a 150 μM solution of Yb^3+^[Zn(ii)MC_pyzHA_] over 15 min followed by illumination with UV-A light (selected with a 377 nm bandpass 50 nm filter) for 8 min and additional incubation for 1 h. The treated cells were then washed and incubated with a 3 μM solution of PI for 5 min. (Bottom) Untreated cells as a control. (A) Brightfield image. (B) NIR emission signal arising from the Yb^3+^[Zn(ii)MC_pyzHA_] MC (*λ*_ex_: 447 nm bandpass 60 nm filter, *λ*_em_: longpass 805 nm filter, exposure time: 8 s). (C) Visible fluorescence signal arising from PI (*λ*_ex_: 550 nm bandpass 25 nm filter, *λ*_em_: 605 nm bandpass 70 nm filter, exposure time: 800 ms). (D) Merged images of (B) and (C). (E) Merged images of (A), (B) and (C). 40× objective.

A novel and useful photochemical phenomenon was observed as a result of the exposure of HeLa cells pre-incubated with a concentrated (>150 μM) solution of Ln^3+^[Zn(ii)MC_pyzHA_] (Ln^3+^ = Yb, Nd) to UV-A light (selected with a 377 nm bandpass 50 nm filter) (Fig. 2A and S1A†). The HeLa cells were fixed and, moreover, after additional incubation time, their nucleus and cytoplasm were stained with Ln^3+^[Zn(ii)MC_pyzHA_] (Fig. 2B and S1B†). The cell fixation process was confirmed by systematic monitoring of the cell morphology over one month by acquiring brightfield microscopic images. No significant changes could be observed during this period of time, indicating the high efficiency and quality of such fixation to preserve the cell morphology (Fig. S2†).

HeLa cells stained with the Yb^3+^[Zn(ii)MC_pyzHA_] complex emit a sufficiently intense signal in the NIR region so that it can be detected not only with our specialized high sensitivity EMCCD camera (Electron Multiplying Charged Coupled Device, Photometrics Evolve 512, Roper Scientific) optimized for NIR imaging but also with a standard CCD commonly installed on fluorescence microscopes (ORCA-R2, Hamamatsu) (Fig. S3A†). In addition, the NIR signal arising from Yb^3+^[Zn(ii)MC_pyzHA_] in HeLa cells could be specifically collected using a 70 nm bandpass filter centred at 980 nm (Fig. S3B†). In contrast, the Nd^3+^ signal arising from HeLa cells stained with Nd^3+^[Zn(ii)MC_pyzHA_], observable with the EMCCD camera, was not sufficiently intense to allow for satisfactory detection either with the standard CCD camera described previously or with bandpass filters (895 nm bandpass 90 nm or 1080 nm bandpass 100 nm). We have, therefore, chosen the Yb^3+^[Zn(ii)MC_pyzHA_] MC to perform further imaging experiments.

In addition, since the permeabilization of cell membranes (plasma and nuclear) is crucial to allow for the specific targeting of cell components, incubation with the commercially available non-permeable nuclear counter stain, propidium iodide (PI), was performed. Results of this experiment indicate the successful permeabilization of both cellular membranes. Moreover, comparable labelling of the nucleus with PI and with Yb^3+^[Zn(ii)MC_pyzHA_] could be observed, suggesting their colocalization (Fig. 2C and D). For all experiments where such simultaneous labelling was obtained, the NIR emission signal arising from Yb^3+^[Zn(ii)MC_pyzHA_] was recorded prior to the addition of PI to avoid any risk of detection of the residual PI emission in the NIR region after longer exposure to the excitation light during image acquisition.

In order to optimize the experimental conditions for the fixation of cells, changes occurring in the cell morphology were monitored with brightfield and NIR luminescence images collected upon the variation of (i) the concentration of Yb^3+^[Zn(ii)MC_pyzHA_] and (ii) the exposure time of the samples to UV-A light. Other parameters, *i.e.* the pre-incubation time (15 min) and the incubation step after cell fixation (1 h), were kept constant.

**Fig. 3:**
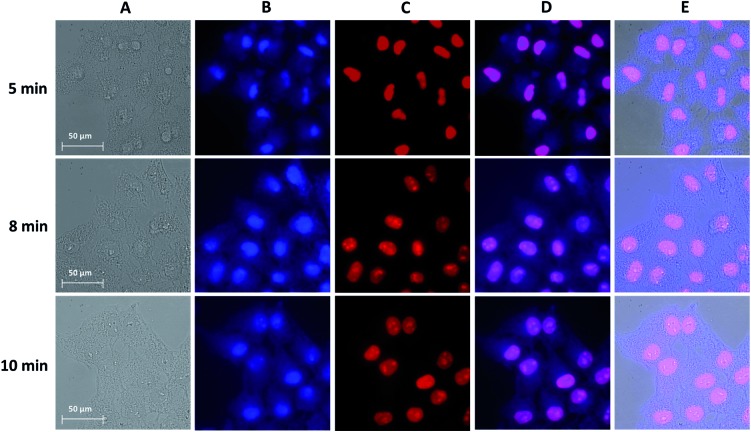
Images obtained from epifluorescence microscopy experiments conducted on HeLa cells incubated with a 150 μM solution of Yb^3+^[Zn(ii)MC_pyzHA_] for 15 min, followed by illumination with UV-A light (selected with a 377 nm bandpass 50 nm filter) for different amounts of time: (top) 5 min, (middle) 8 min and (bottom) 10 min, and further incubation for 1 h. The treated cells were then washed and incubated with a 3 μM solution of PI for 5 min. (A) Brightfield. (B) NIR signal arising from Yb^3+^[Zn(ii)MC_pyzHA_] (*λ*_ex_: 447 nm bandpass 60 nm filter, *λ*_em_: longpass 805 nm filter, exposure time: 8 s). (C) Visible signal arising from PI (*λ*_ex_: 550 nm bandpass 25 nm filter, *λ*_em_: 605 nm bandpass 70 nm filter, exposure time: 800 ms). (D) Merged images of (B) and (C). (E) Merged images of (A), (B) and (C). 63× objective.

For the first series of experiments, the concentration of Yb^3+^[Zn(ii)MC_pyzHA_] was maintained at 150 μM. We observed that the morphologies of living cells were preserved for the cells fixed by exposure to UV-A light for 8 and 10 min, while shorter illumination times had a significant impact on cell morphologies (Fig. 3A). Nevertheless, in all cases, the NIR signal could be unambiguously detected (Fig. 3B). From this series of results, we can conclude that illumination for 8 or 10 min is the best condition for the fixation of HeLa cells incubated with a 150 μM solution of Yb^3+^[Zn(ii)MC_pyzHA_].

Secondly, to test whether lower concentrations of Yb^3+^[Zn(ii)MC_pyzHA_] can be used for the design of experiments combining cell fixation and NIR cellular staining, the brightfield and NIR luminescence images obtained from HeLa cells incubated with a 15 μM, 30 μM or 60 μM solution of the MC were acquired after 8 min of illumination with UV-A light (Fig. S4†). For the 15 μM concentration, the cell morphology was disrupted and its fixation was not observed, since the cells were detached from the growth support 3 hours after the experiment. HeLa cells incubated with a 30 or 60 μM solution of Yb^3+^[Zn(ii)MC_pyzHA_] were fixed but the staining of the nucleus and the cytoplasm was not observed, an effect which could be attributed to insufficient concentration of the lanthanide(III) NIR-emitting counter stain.

Accordingly, the optimized experimental conditions to obtain the combined NIR staining and fixation of HeLa cells while preserving their morphology are: pre-incubation for 15 min with a 150 μM solution of Yb^3+^[Zn(ii)MC_pyzHA_], followed by 8 min of illumination with UV-A light, and further incubation for 1 h. It should be noted here that the incubation of HeLa cells with a 150 μM solution of Yb^3+^ MC for a longer amount of time (12 h) without illumination is toxic for cells and leads to their death (Fig. S5A†). Nevertheless, epifluorescence microscopy experiments confirmed that the NIR emission can be unambiguously detected in cells incubated under these experimental conditions (Fig. S5B†), and strongly suggest the colocalization of the signals arising from Yb^3+^[Zn(ii)MC_pyzHA_] in the NIR region, and the nuclear stain, PI, in the visible region.

**Fig. 4:**
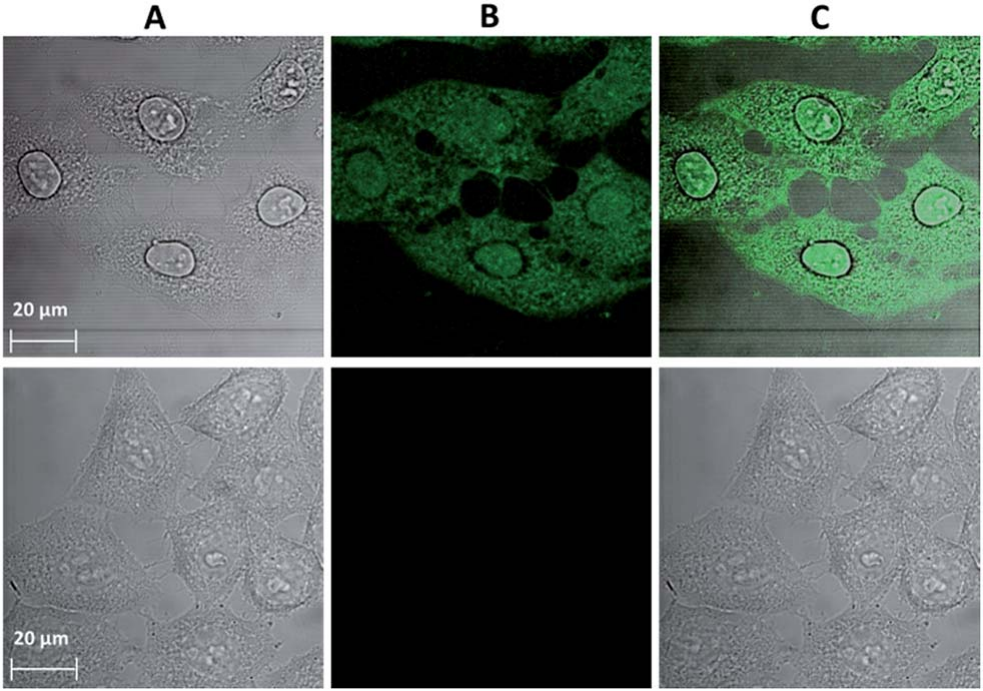
Images obtained from confocal microscopy experiments performed on HeLa cells: (top) incubated with a 150 μM solution of Yb^3+^[Zn(ii)MC_pyzHA_] for 15 min followed by illumination with UV-A light (selected with a 377 nm bandpass 50 nm filter) for 8 min and further incubation for 1 h (*λ*_ex_: 458 nm, *λ*_em_: 499–799 nm, 63× objective, 2× zoom). (Bottom) Untreated cells as a control (63× objective, 1.5× zoom). (A) Brightfield image. (B) Signal arising from Yb^3+^[Zn(ii)MC_pyzHA_] in the visible region. (C) Merged images of (A) and (B).

Confocal microscopy is an essential tool for optical imaging as its increased resolution allows for the collection of information about the precise localization of the luminescence probes. However, due to the current lack of confocal microscopes with a detection ability in the NIR range, the emission signal arising from the chromophoric MC scaffold/ligands in the visible range was acquired for the cells incubated with Yb^3+^[Zn(ii)MC_pyzHA_] under the optimized experimental conditions described previously, allowing for the combined monitoring of cell fixation and staining (Fig. 4). It was confirmed that the Yb^3+^ MCs are located both in the nucleus and in the cytoplasm of the HeLa cells. The possibility to detect signals in the visible region, in addition to the NIR emission of Yb^3+^, broadens the potential applications of Yb^3+^[Zn(ii)MC_pyzHA_] to include multiplex biological imaging, and decreases the risk of false negative or false positive results.

In order to evaluate the influence of UV-A light on the photophysical properties of Yb^3+^[Zn(ii)MC_pyzHA_] and Nd^3+^[Zn(ii)MC_pyzHA_], excitation/emission spectra and quantitative parameters such as the Ln^3+^-centred quantum yields and luminescence lifetimes were measured under biological conditions mimicking the ones used for microscopic experiments, *i.e.* cell culture media (Opti-MEM + 2% FBS) with and without exposure to UV-A light. The same type of study was also performed on a suspension of HeLa cells treated with Yb^3+^ or Nd^3+^ MCs under the optimized experimental conditions determined and described previously, leading to combined fixation and staining (for additional information about the sample preparation see the experimental section). Unfortunately, the experiments performed on the suspension of HeLa cells stained with the Nd^3+^ MC revealed that the signal was too faint for the acquisition of emission/excitation spectra or for the measurement of the quantum yield. However, luminescence decay curves could be collected, as a laser system was used for the excitation of the MCs in this type of experiment.

**Fig. 5:**
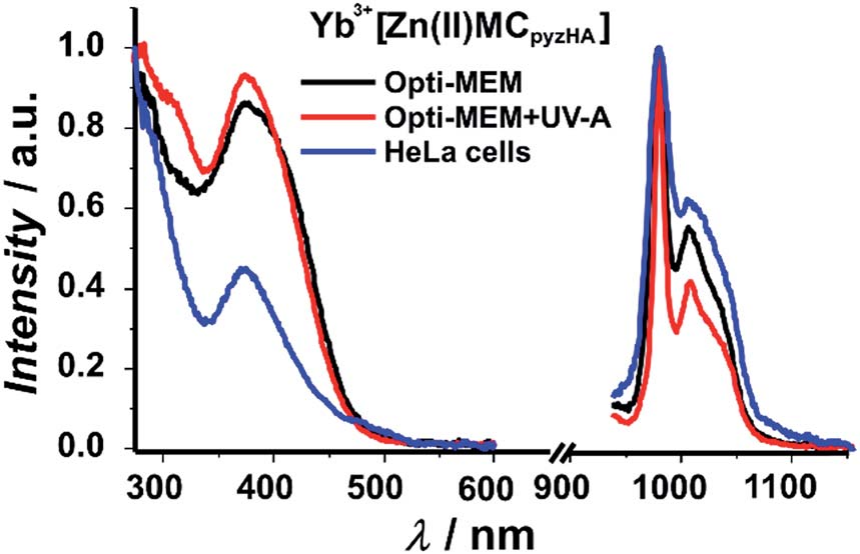
Excitation (left plots: *λ*_em_ = 980 nm) and emission (right plots: *λ*_ex_ = 370 nm) spectra of a 150 μM solution of Yb^3+^[Zn(ii)MC_pyzHA_] in cell culture medium (Opti-MEM + 2% FBS) with or without exposure to UV-A light, and of a suspension of HeLa cells stained with the Yb^3+^ MC at room temperature.

Excitation spectra of the Yb^3+^ and Nd^3+^ MCs, collected upon monitoring the ^2^F_5/2_ → ^2^F_7/2_ or ^4^F_3/2_ → ^4^I_11/2_ transitions in the NIR region that are centred at 980 and 1070 nm, respectively, exhibit broad ligand-centred bands in the UV and visible regions up to 500 nm (Fig. 5 and S6†). As the Yb^3+^ cation does not possess electronic levels located at energies corresponding to the UV or visible regions, the presence of this broad band in these spectral domains indicates that the sensitization of Yb^3+^ has to be associated with energy transfer from the MC scaffold. The high similarity of the excitation spectra of Ln^3+^[Zn(ii)MC_pyzHA_] with Ln^3+^ = Yb and Nd indicates that the sensitization of both NIR emitting lanthanide cations is taking place following the same energy paths. We also observe in the excitation spectra of each of these MCs the presence of intra-ligand charge transfer bands, with maxima located around 370 nm, that are specific to the Zn_16_Ln MC structure. This observation strongly suggests that the complexes remain intact under different biological conditions including when located in HeLa cells. Upon excitation into the ligand-centred bands at 370 nm, the Yb^3+^ and Nd^3+^ MCs exhibit characteristic emissions in the NIR region arising from f–f transitions (Fig. 5 and S6†).

Experimental luminescence decays of both the Yb^3+^ and Nd^3+^ MCs in cell culture media were best fitted by biexponential functions with short (*τ*_1_) and long (*τ*_2_) components, indicating that the luminescent Ln^3+^ complexes are affected by the presence of different (bio)molecules under the chosen experimental conditions, and are located in two different microenvironments (Table 1). It should be recognized that the luminescence decays of Ln^3+^[Zn(ii)MC_pyzHA_] (Ln^3+^ = Nd, Yb) in the solid state and in aqueous solutions could be perfectly fitted with a monoexponential function, reflecting the presence of a unique well-defined environment around Ln^3+^.^27^ The *τ*_1_ value is comparable with the lifetime obtained for the solutions of Ln^3+^[Zn(ii)MC_pyzHA_] MCs in water, while the *τ*_2_ value is 3–4 times longer (Table 1). The illumination of the solutions of Ln^3+^[Zn(ii)MC_pyzHA_] in cell culture media with UV-A light leads to an increase of the *τ*_2_ contribution to the luminescence decay by a factor of 3–4. Such results emphasize the importance of UV-A light for the photophysical properties of Ln^3+^[Zn(ii)MC_pyzHA_] in cell culture media. The luminescence decays recorded on suspensions of HeLa cells stained with Yb^3+^ or Nd^3+^ MCs are exclusively monoexponential, with luminescence lifetimes comparable to the corresponding long *τ*_2_ component described previously. The lengthening of the *τ* values obtained for the solutions of Ln^3+^[Zn(ii)MC_pyzHA_] in cell culture media and in stained HeLa cells, compared to the values observed in water, indicates an improved level of protection of both Ln^3+^ cations against sources of non-radiative deactivation. This change in the MC environment possibly is the result of alterations in the second coordination sphere of Ln^3+^, *i.e.* a partial or complete replacement of H_2_O molecules by less quenching (bio)molecules that are facilitated by the impact of UV-A light.

**Table 1:** Photophysical properties of the Yb^3+^[Zn(ii)MC_pyzHA_] and Nd^3+^[Zn(ii)MC_pyzHA_] MCs obtained under different biological conditions at room temperature^*a*^

Metallacrown	Condition	*Q* ^*c*^ (%)	*τ*_1_ ^*d*^ (μs)	*τ*_2_ ^*d*^ (μs)	<*τ*>^*e*^ (μs)
Yb^3+^[Zn(ii)MC_pyzHA_]	200 μM in H_2_O^*b*^	1.12(7) × 10^–2^	5.57(1): 100%		
	150 μM in ‘Opti-MEM + 2% FBS’	1.25(1) × 10^–2^	6.45(5): 89%	24(1): 11%	12(1)
	150 μM in ‘Opti-MEM + 2% FBS’ + UV-A	2.05(5) × 10^–2^	7.03(3): 61%	21.5(1): 39%	16.6(8)
	Suspension of stained HeLa cells	2.14(6) × 10^–2^		23.6(7): 100%	
Nd^3+^[Zn(ii)MC_pyzHA_]	200 μM in H_2_O^*b*^	7.7(1) × 10^–3^	0.214(4): 100%		
	150 μM in ‘Opti-MEM + 2% FBS’	7.50(5) × 10^–3^	0.256(6): 90%	1.1(1): 10%	0.5(1)
	150 μM in ‘Opti-MEM + 2% FBS’ + UV-A	7.7(1) × 10^–3^	0.352(2): 67%	0.874(7): 33%	0.641(9)
	Suspension of stained HeLa cells	n.d.		0.808(8): 100%	

^*a*^2*σ* values are given between parentheses. Experimental errors: *τ*, ±2%; *Q*, ±10%.

^*b*^Data from ref. 27.

^*c*^Under excitation at 370 nm.

^*d*^Under excitation at 355 nm. Percentages of the contributions of each individual lifetime value are provided after the colon.

^*e*^Average lifetime.

The quantum yield (*Q*) values recorded under excitation of the ligand-centred bands (370 nm) of Yb^3+^[Zn(ii)MC_pyzHA_] in a cell culture medium that have been illuminated with UV-A light and of a suspension of stained HeLa cells are 1.7–1.9 times larger than the ones observed in water or in Opti-MEM + 2% FBS without illumination with UV-A light (Table 1). Such differences in the *Q* values are in line with the changes in the Yb^3+^ luminescence lifetimes and the decrease in the contribution of non-radiative quenching processes. Surprisingly, the Nd^3+^ MC does not show the same trend, as the recorded quantum yield values are similar under all tested conditions. The rationalization of this observation requires further investigation.

**Fig. 6:**
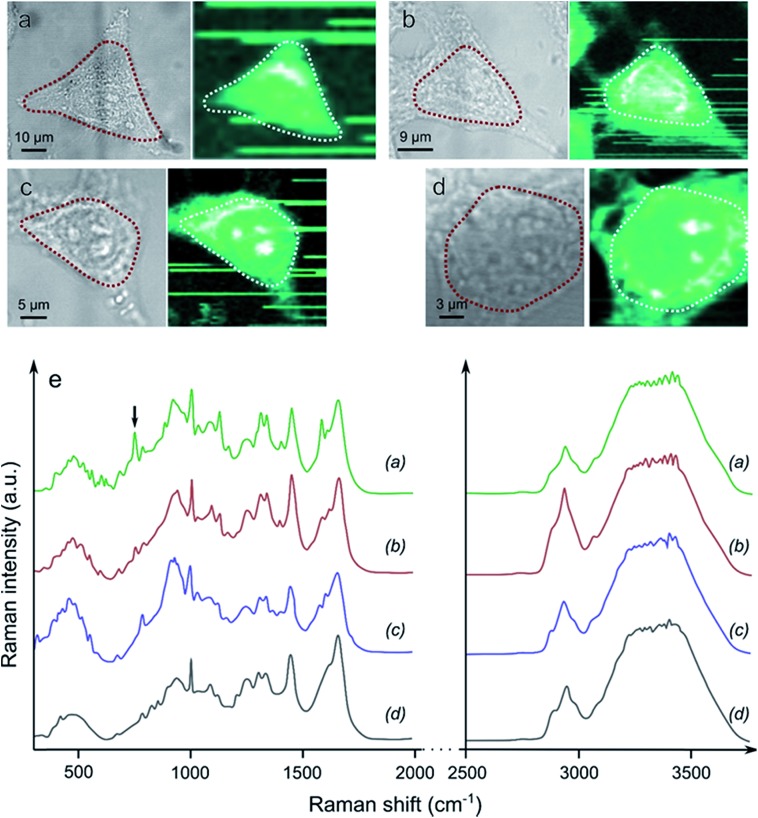
Brightfield images and associated Raman spectroscopy mapping of the CH band intensities of (a) a living cell, and a cell fixed with (b) PFA, (c) methanol and (d) Yb^3+^[Zn(ii)MC_pyzHA_]. The areas surrounded by dashed lines correspond to the maximal intensities of the CH Raman signals. (e) The associated averaged Raman spectra. The black arrow at 752 cm^–1^ points to the position of the band attributed to cytochrome c.

Raman spectroscopy mapping experiments were performed in order to compare the qualities of the cell fixation processes obtained with Yb^3+^[Zn(ii)MC_pyzHA_] in comparison to classical fixation methods based on paraformaldehyde (PFA) or methanol (Fig. 6). The ability of Raman spectroscopy to discriminate between living and fixed cells has been demonstrated previously.^28,29^ More specifically, the decrease of the 752 cm^–1^ peak corresponding to a vibration of cytochrome c has been unambiguously observed after the fixation of living cells with PFA.^29^ Accordingly, a set of vibrational frequencies (a cell fingerprint) corresponding to specific chemical bonds^30^ was recorded from the cells that were fixed with Yb^3+^[Zn(ii)MC_pyzHA_] and by the classical methods described above. Signals were collected in the range of 0–4000 cm^–1^ in different cellular compartments (the cytoplasm and nucleus), allowing for the monitoring of the variations of specific vibrations arising from the nucleic acids and proteins. Particular interest was focused on the CH vibrational bands at 2800–3300 cm^–1^ that reflect a distribution of proteins, lipids and carbohydrates in cells, which are typically used for the localization of cellular organelles, as well as the OH bands in the range of 3100–3650 cm^–1^.^31^ The averaged Raman spectrum corresponding to the area of the cell with the highest intensity of the CH band signal (*e.g.* the cytoplasm and nucleus, dashed lines in Fig. 6a–d) was extracted for each sample. Highly similar biomolecular profiles were observed for living HeLa cells and those fixed with Yb^3+^[Zn(ii)MC_pyzHA_], PFA or methanol. The only pronounced difference, which was detected spectrally in each case, is the decrease of the 752 cm^–1^ peak intensity, corresponding to cytochrome c, for the fixed cells compared to the living ones. The same effect has been previously reported for the fixation with PFA.^29^ A more detailed and complex analysis of the Raman spectra is required in order to decipher the exact effect induced by different fixation techniques on cellular components.

## Conclusion

We have demonstrated in this work the unique ability of NIR-emitting Zn^2+^/Ln^3+^ metallacrowns, Ln^3+^[Zn(ii)MC_pyzHA_], to act as combined cell fixation and NIR emission based whole-cell counter staining agents upon an initial short exposure to UV-A light.

There is only one similar example described in the literature of such combined fixation and staining, which was reported for the Xenopus XTC-2 nucleus with the organic imaging agent Hoechst 33342. This molecule emits in the visible region upon illumination with UV light at 365 nm. In contrast, Ln^3+^[Zn(ii)MC_pyzHA_] simultaneously stains and fixes the nucleus and cytoplasm of HeLa cells. Another level of novelty provided by this work is that both Nd^3+^ and Yb^3+^ MCs generate a long term photostable emission in the NIR region. The luminescence in this spectroscopic domain is highly advantageous for biological optical imaging applications due to the reduced impact of the autofluorescence, allowing for less ambiguous, more precise and sensitive detection that may be quantified over long periods. In addition, counter staining with the Yb^3+^ and Nd^3+^ MCs results in the generation of characteristic atom-like sharp emission bands in the NIR region, minimizing their overlap with the ones of commercially available probes used for the specific labelling of cell components, thus simplifying the interpretation of the results and increasing the reliability of analysis. It should be noted here that there is one example in the literature where signals from Nd^3+^ and Yb^3+^ complexes could be detected with a scanning confocal microscope which was not adapted for imaging in the NIR range.^32^ We have been able to show here that due to the unusually high brightness of Yb^3+^[Zn(ii)MC_pyzHA_], cellular images could be acquired on standard epifluorescence microscopes without using any specialized equipment optimized for detection in the NIR region.

The long-term ability of the proposed fixation technique to preserve cell morphology has been confirmed by continuous monitoring of the brightfield images, while the similarity of the biomolecular profiles of cells fixed by Ln^3+^[Zn(ii)MC_pyzHA_] and traditional methods with PFA or methanol has been confirmed by Raman spectroscopy.

As another achievement that is part of this work, we have developed a methodology and performed a detailed analysis of the photophysical properties (emission/excitation spectra and luminescence quantum yields and lifetimes) of Ln^3+^[Zn(ii)MC_pyzHA_] in aqueous solution and under conditions mimicking the ones used for microscopic experiments, including in a suspension of stained HeLa cells. Such studies allowed us to establish that the MC structure remains intact in various environments. On the other hand, the presence of (bio)molecules in cell culture media and illumination with UV-A light have a pronounced impact on the luminescence lifetimes and quantum yields of Ln^3+^[Zn(ii)MC_pyzHA_], causing them to increase. These results indicate that the photophysical parameters collected in aqueous solutions are not always representative of the behaviour of luminescence probes in real biological systems, and demonstrate the necessity to perform more extensive studies to assess how such a molecular system will behave.
