# Supplementary material for: Near-infrared luminescent metallacrowns for combined in vitro cell fixation and counter staining
Source: Chem Sci. 2017 Aug 8;8(9):6042–50. doi: 10.1039/c7sc01872j (PMC5625569; doi:10.1039/c7sc01872j)
Supplement: Supplementary file 1 [file SC-008-C7SC01872J-s001.pdf]

Electronic Supplementary Material (ESI) for Chemical Science.
This journal is © The Royal Society of Chemistry 2017

Electronic Supplementary Information (ESI)

Near-Infrared Luminescent Metallacrowns for Combined *in vitro* Cell Fixation and Counter Staining

Ivana Martinić[a], Svetlana V. Eliseeva[a]*, Tu N. Nguyen[b], Frédéric Foucher[a], David Gosset[a], Frances Westall[a], Vincent L. Pecoraro[b]*, Stéphane Petoud[a]‡*

[a] Centre de Biophysique Moléculaire, CNRS UPR 4301, 45071 Orléans Cedex 2, France

[b] Department of Chemistry, Willard H.Dow Laboratories, University of Michigan, 930 N. University Ann Arbor, Michigan 48109, United States

‡ Current address: Department of Inorganic, Analytical and Applied Chemistry, University of Geneva, CH-1211 Geneva 4, Switzerland

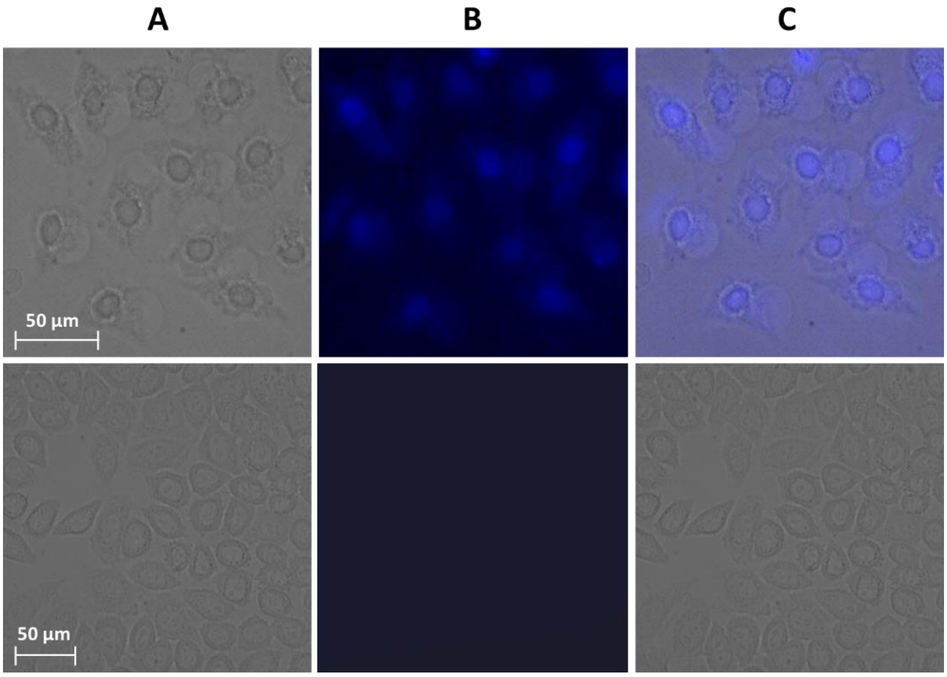

Figure S1. HeLa cells pre-incubated with 150 µM of Nd^{3+}[Zn(II)MC_{pyzHA}] during 15 min followed by an illumination with UV-A light (377 nm band pass 50 nm filter) during 8 min and further incubation during 1 h. (A) Brightfield. (B) NIR signal arising from Nd^{3+}[Zn(II)MC_{pyzHA}] (λ_{ex}: 377 nm band pass 50 nm filter, λ_{em}: long pass 805 nm filter, exposure time: 12s) (C) Merged between (A) and (B). 40× objective.

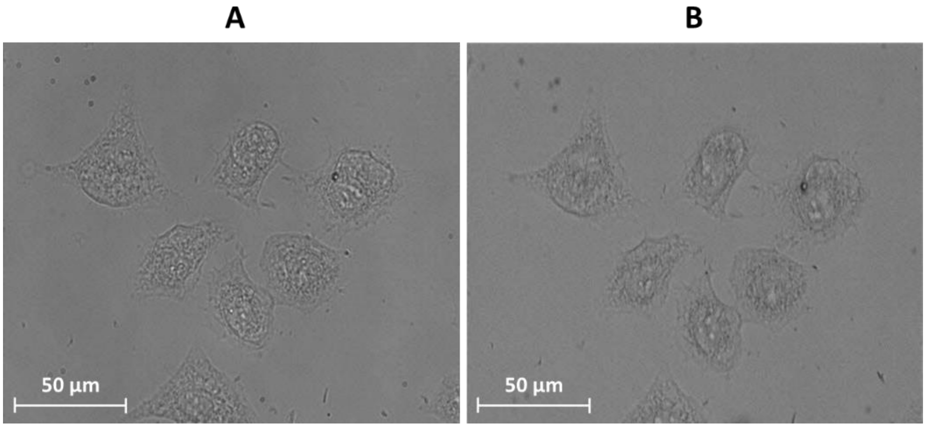

Figure S2. Brightfield images of HeLa cells fixed with Yb^{3+}[Zn(II)MC_{pyzHA}] (150 µM, pre-incubation: 15 min, illumination with UV-A light: 8 min, incubation: 1h) and recorded after different storage times at 37°C in Opti-MEM media: (A) 1 h and (B) 1 month.

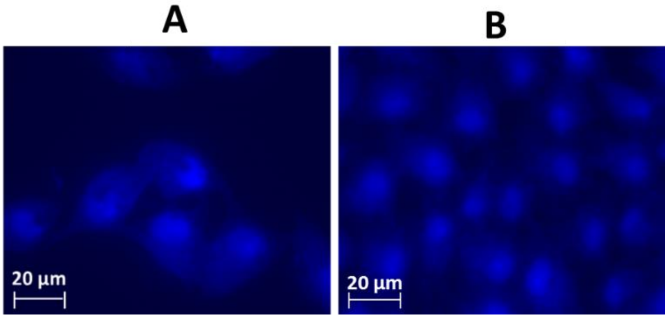

Figure S3. Images obtained from the epifluorescence microscopy experiments performed on fixed HeLa cells treated with Yb^{3+}[Zn(II)MC_{pyzHA}]. NIR emission signal was detected with the standard CCD camera (Hamamatsu ORCA-R2). (A) λ_{ex}: 447 nm band pass 60 nm filter, λ_{em}: long pass 805 nm filter, exposure time: 30s. (B) λ_{ex}: 447 nm band pass 60 nm filter, λ_{em}: 996 nm band pass 70, exposure time: 80s. 63× objective.

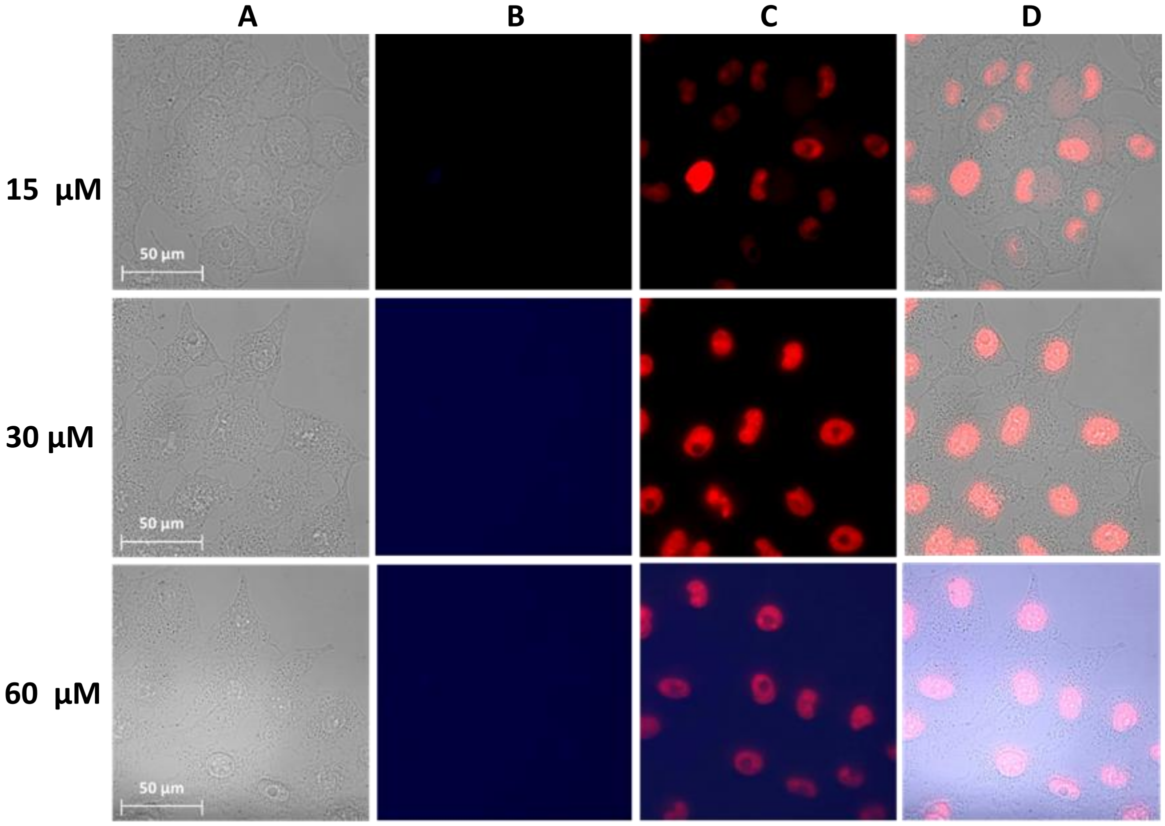

Figure S4. Images obtained from the epifluorescence microscopy experiments performed on HeLa cells incubated with a (top) 15 µM, (middle) 30 µM or (bottom) 60 µM solution of Yb^{3+}[Zn(II)MC_{pyzHA}] during 15 min, followed by an illumination with UV-A light (377 nm band pass 50 nm filter) during 8 min, and further incubation during 1 h. Treated cells were washed and incubated with 3 µM solution of PI during 5 min. (A) Brightfield. (B) NIR signal arising from Yb^{3+}[Zn(II)MC_{pyzHA}] (λ_{ex}: 447 nm band pass 60 nm filter, λ_{em}: long pass 805 nm, exposure time: 8s). (C) Visible fluorescence signal arising from PI (λ_{ex}: 535 nm band pass 40 nm filter, λ_{em}: 617 nm band pass 40 nm filter, exposure time: 800 ms). (D) Merged image between (B) and (C). (E) Merged image between (A), (B) and (C). 63× objective.

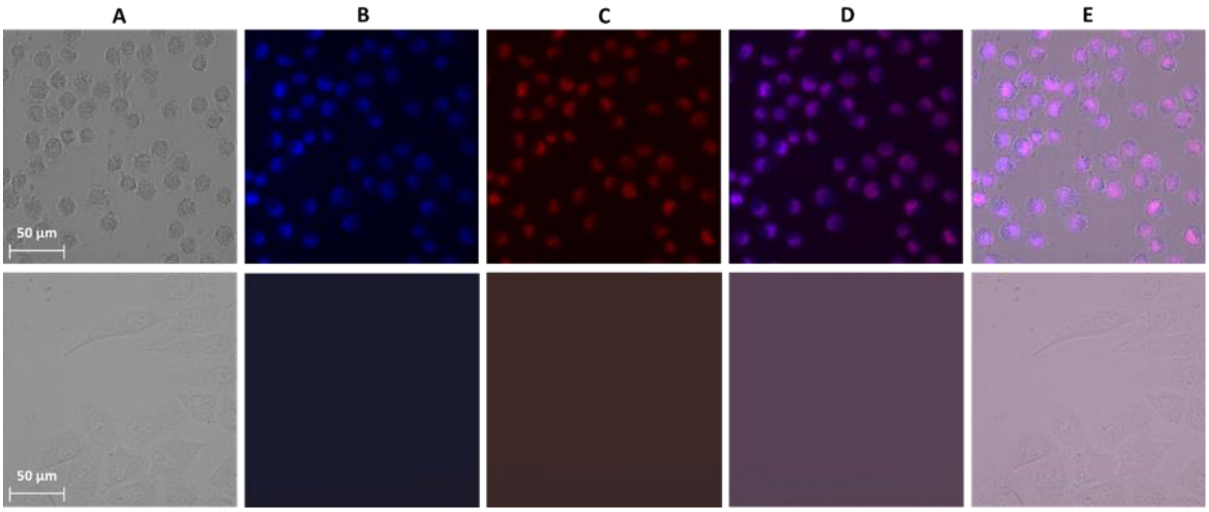

Figure S5. Images obtained from the epifluorescence microscopy experiments performed on HeLa cells. (Top) Incubated with a 150 µM solution of Yb^{3+}[Zn(II)MC$_{pyzHA}$] during 12h, washed and incubated with a 3 µM solution of PI during 5 min. (Bottom) Untreated cells as control. (A) Brightfield. (B) NIR signal arising from Yb^{3+}[Zn(II)MC$_{pyzHA}$] (λ_{ex}: 447 nm band pass 60 nm filter, λ_{em}: long pass 805 nm filter, exposure time: 5s). (C) Visible fluorescence signal arising from PI (λ_{ex}: 535 nm band pass 40 nm filter, λ_{em}: 617 nm band pass 40 nm filter, exposure time: 800 ms). (D) Merged image between (B) and (C). (E) Merged image between (A), (B) and (C). 63× objective.

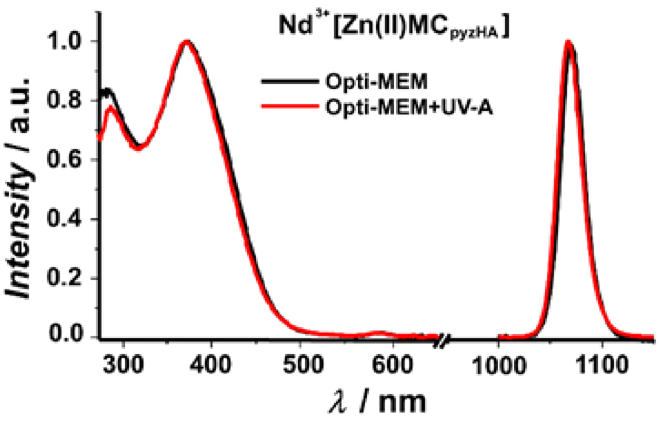

Figure S6. Excitation (left plots : λ_{em}= 1070 nm) and emission (right plots: λ_{ex} = 370 nm) spectra of a 150µM solution of Nd^{3+}[Zn(II)MC$_{pyzHA}$] in cell culture media (Opti-MEM + 2% FBS) with or without exposure to the UV-A light at room temperature.
